# The efficacy and safety of PD-1 inhibitors combined with chemotherapy treatment for advanced esophageal cancer: a network meta-analysis

**DOI:** 10.3389/fmed.2024.1515263

**Published:** 2025-01-10

**Authors:** Jian-Zhou Tian, Li Zhang, Fu-Yong Lin, Ren-Jiao He, Wen-Rong Tian, Liu Yan, Guo-Xin Huang, Jin-Wei Ai, Bin Pei, De-Sheng Li

**Affiliations:** ^1^Evidence-Based Medicine Center, Xiangyang No.1 People’s Hospital, Hubei University of Medicine, Xiangyang, China; ^2^Department of Central Sterile Supply, Fujian Medical University Union Hospital, Fuzhou, China; ^3^Department of Plastic Surgery and Regenerative Medicine, Fujian Medical University Union Hospital, Fuzhou, China; ^4^Department Three of Orthopedics/Plastic Surgery, Xiangyang No.1 People’s Hospital, Hubei University of Medicine, Xiangyang, China

**Keywords:** advanced esophageal cancer, PD-1 inhibitors, chemotherapy, combined therapy, network meta-analysis

## Abstract

**Objective:**

This study systematically evaluated the efficacy of programmed death 1 (PD-1) inhibitors combined with chemotherapy for advanced esophageal cancer (EC).

**Methods:**

PubMed, Embase, Web of Science, Scopus, and Cochrane Library were searched to identify related randomized controlled trials (RCTs).

**Results:**

Seven RCTs involving 4,363 participants were included. The results of the direct comparison showed that, compared with chemotherapy alone, PD-1 inhibitors combined with chemotherapy significantly improved overall survival (OS) (HR = 0.69, 95%CI = 0.63–0.74), progression-free survival (PFS) (HR = 0.63, 95%CI = 0.58–0.67), objective response rate (ORR) (RR = 1.41, 95%CI = 1.28–1.57), but were associated with a slight increase in treatment-related adverse events (AEs) (RR = 1.08, 95%CI = 1.03–1.14). The results of the network meta-analysis showed that toripalimab, sintilimab or camrelizumab, and nivolumab combined with chemotherapy were the best in OS, PFS, and ORR, respectively, with camrelizumab showing the lowest incidence of AEs.

**Conclusion:**

These results suggest that PD-1 inhibitors combined with chemotherapy provide superior clinical benefits over chemotherapy alone, albeit with a moderate increase in AEs. However, further verification through multi-center, high-quality RCTs with larger sample sizes is needed to confirm these findings.

**Systematic review registration: https://www.crd.york.ac.uk/prospero/display_record.php?ID=CRD42024627485.:**

## Introduction

Esophageal cancer (EC) is the seventh most common cancer worldwide, with over 600,000 new cases and 540,000 deaths annually (1). It primarily affects the upper digestive tract and is classified into squamous cell carcinoma (ESCC) and adenocarcinoma, with ESCC accounting for approximately 70–80% (2). Conventional treatment options, including radiotherapy, chemotherapy, surgical resection, or their combinations, have shown limited efficacy (3). Due to late-stage diagnosis in most patients, where the disease has often metastasized to surrounding tissues or organs, the 5-year survival rate remains as low as 20% (4).

Programmed death 1 (PD-1) inhibitors offer a novel therapeutic approach by counteracting tumor-induced T-cell inhibition through blockade of the PD-1/programmed death ligand 1 or 2 (PD-L1/PD-L2) pathway. This restores T-cell activity, enhances tumor antigen expression, and improves tumor cell killing (5, 6). When combined with chemotherapy, PD-1 inhibitors have demonstrated synergistic effects, further enhancing therapeutic outcomes (7, 8). Pembrolizumab, for instance, has recently been approved by the FDA as a second-line treatment for advanced ESCC (9). Additionally, the combination of PD-1 inhibitors and chemotherapy has shown efficacy across multiple malignancies, including, but not limited to, triple-negative breast cancer, advanced non-small cell lung cancer, advanced melanoma, advanced gastric cancer, and Hodgkin lymphoma (10–14).

Clinical randomized controlled trials (RCTs) have indicated that nivolumab or pembrolizumab combined with fluorouracil/cisplatin has improved overall survival (OS) and progression-free survival (PFS) compared to fluorouracil/cisplatin alone, suggesting their potential as first-line treatments for advanced EC (15–21). However, there is limited evidence directly comparing the efficacy and safety of various PD-1 inhibitors combined with chemotherapy regimens in this context. To address this gap, we conducted a network meta-analysis to systematically evaluate and rank different PD-1 inhibitor-based regimens based on therapeutic efficacy and safety, providing robust evidence to inform clinical decision-making for advanced EC treatment.

## Methods

This study was reported in accordance with PRISMA guidelines (22). And the study was registered in Prospero (CRD42024627485).

### Literature search strategy

We conducted a comprehensive search of PubMed, Embase, Web of Science, Scopus, the Cochrane Library (Issue 2, 2024), and clinicaltrials.gov to identify RCTs assessing the efficacy and safety of PD-1 inhibitor combined with chemotherapy compared to chemotherapy alone in advanced EC. The search spanned from databases inception to February 12, 2024. A combination of Medical Subject Headings (MeSH) terms and free-text keywords was used, including terms such as PD-1 inhibitors, immune check blockade, PD-1, PD-L1, drug therapy, chemotherapy, EC, and so on. Search strategies were tailored for each database and are detailed in Supplementary File S1.

### Inclusion criteria

Study Type: RCTs about PD-1 inhibitors combined with chemotherapy treatment for advanced EC. Participants: Patients diagnosed with advanced EC confirmed by histological or cytological examination. Intervention and Comparison: Studies comparing PD-1 inhibitor combined with chemotherapy against chemotherapy alone. The chemotherapy used in both groups adhered to first-line drug treatments based on NCCN Clinical Practice Guidelines in Oncology (23). Outcomes: Reported OS, PFS, objective response rate (ORR), and treatment-related adverse events (AEs) graded per the National Cancer Institute Common Terminology Criteria for Adverse Events (CTCAE) version 5.0 (24).

### Exclusion criteria

Studies on gastroesophageal junction cancers; studies without extractable data or duplicate publications; non-English studies.

### Literature screening, data extraction, and quality evaluation

Two independent reviewers screened the literature based on predefined inclusion and exclusion criteria, extracted relevant data, and assessed the methodological quality of included studies. Extracted data included general study information (e.g., first author, year of publication, and title of the included studies), patient characteristics (e.g., age, gender, performance status), intervention details, and outcome measures [e.g., hazard ratio (HR) and 95% confidence interval (CI) for OS and PFS, incidence for ORR and AEs]. Risk of bias was evaluated using the Cochrane Risk of Bias Tool, covering random sequence generation, allocation concealment, blinding, incomplete outcome data, selective reporting, and other potential sources of bias. Disagreements were resolved through discussion or consultation with a third reviewer.

### Statistical methods

Stata 14.0 was used for direct meta-analysis. Hazard ratios (HRs) with 95% confidence intervals (CIs) were calculated for OS and PFS, while relative risks (RRs) with 95% CIs were used for ORR and AEs. Heterogeneity was assessed using the I^2^ statistic and Q-test. Fixed-effect models were applied when *I^2^* ≤ 50% and *p* > 0.1; otherwise, random-effect models were used. Subgroup analyses were performed to explore potential effect modifiers, including gender, age, Eastern Cooperative Oncology Group (ECOG) performance score, tumor proportion score (TPS), combined positive score (CPS), and smoking status. Sensitivity analyses were conducted using a leave-one-out approach to ensure robustness. Publication bias was evaluated using funnel plots and Egger tests.

For network meta-analysis, the Gemtc package in R 4.2.2 was employed. Network diagrams for each outcome were constructed, and indirect comparisons were conducted using Bayesian statistical methods with the Markov Chain Monte Carlo (MCMC) fixed-effect model. Relevant parameters were set to four chains, with n.adapt = 20,000 and n.iter = 50,000. Cumulative ranking probabilities and the surface under the cumulative ranking (SUCRA) were calculated to rank the efficacy and safety of interventions.

## Results

### Literature search results

**Figure 1:**
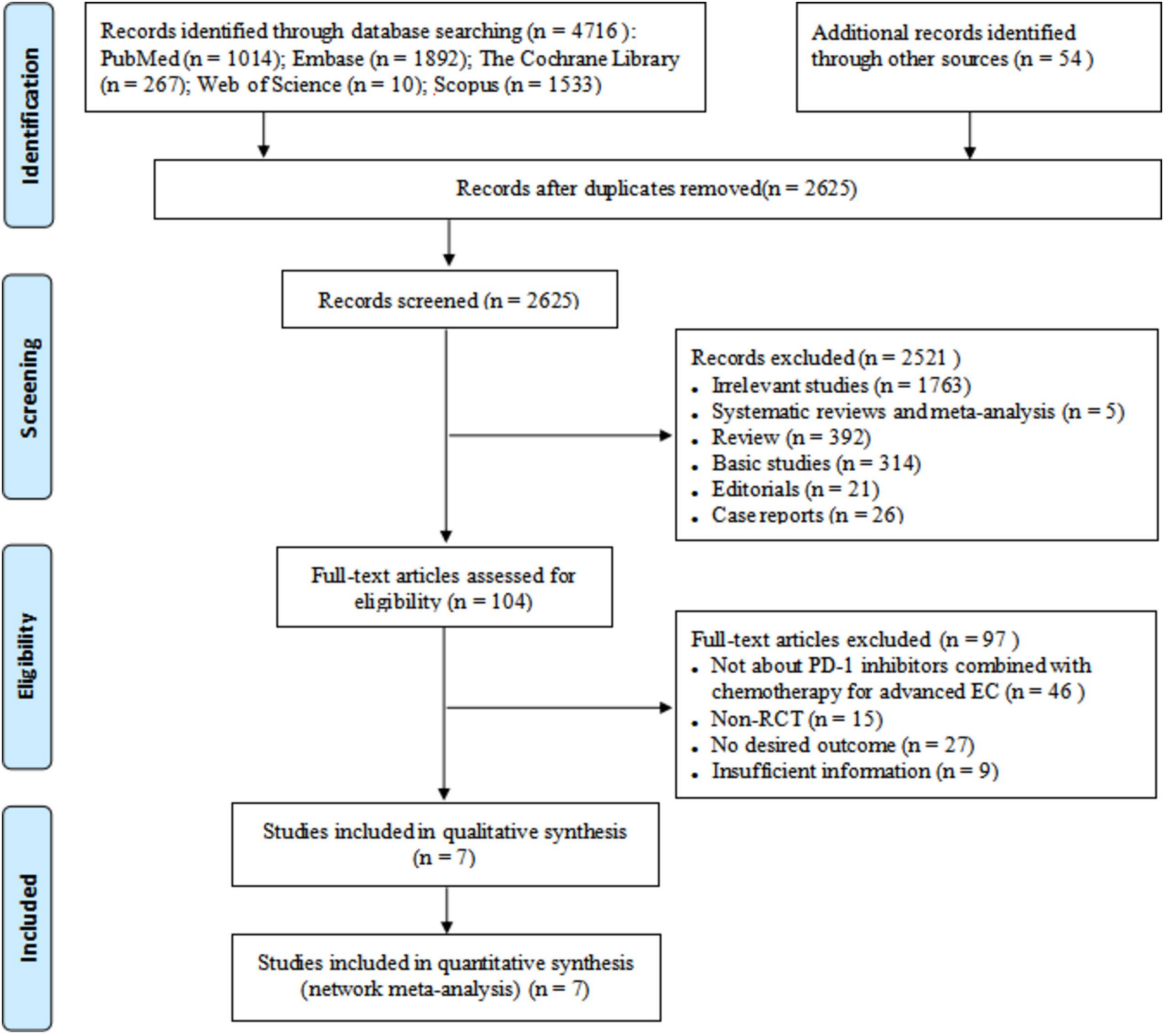
The literature screening process and results.

A total of 4,716 relevant literatures were retrieved, and seven studies were finally included after screening (15–21). The literature screening process and results are shown in Figure 1.

### Basic characteristics included in the study

The basic characteristics of the included studies are shown in Table 1. Seven RCTs involved 4,363 patients with advanced EC, including 2,270 in the PD-1 inhibitor combined chemotherapy group and 2093 in the chemotherapy alone group.

**Table 1:** The basic characteristics of the included studies.

Study	Intervening measure	Number of patients		Median age (years)	ECOG performance status-score		HR for OS
		Male	Female		0	1	
ASTRUM-007 (20)	Serplulimab + chemotherapy	317	51	64	93	275	0.68(0.53–0.87)
	Placebo + chemotherapy	153	30	64	53	130	
CheckMate-648 (18)	Nivolumab + chemotherapy	253	68	64	150	171	0.74(0.61–0.89)
	Chemotherapy	275	49	64	154	170	
ESCORT-1st (16)	Camrelizumab + chemotherapy	260	38	62	71	227	0.70(0.56–0.88)
	Placebo + chemotherapy	263	35	62	66	232	
JUPITER-06 (17)	Toripalimab + chemotherapy	217	40	63	66	191	0.58(0.43–0.78)
	Placebo + chemotherapy	220	37	62	68	189	
KEYNOTE-590 (15)	Pembrolizumab + chemotherapy	306	67	64	149	223	0.73(0.62–0.86)
	Placebo + chemotherapy	319	57	62	150	225	
ORIENT-15 (19)	Sintilimab + chemotherapy	279	48	63	77	250	0.63(0.51–0.78)
	Placebo + chemotherapy	288	44	63	81	251	
RATIONALE-306 (21)	Tislelizumab + chemotherapy	282	44	64	109	217	0.66(0.54–0.80)
	Placebo + chemotherapy	281	42	65	104	219	

ECOG, Eastern Cooperative Oncology Group; The Eastern Cooperative Oncology Group performance status score 5-point scale defines 0 as fully active, able to carry on all predisease performance without restriction, and defines 1 as restricted in physically strenuous activity but ambulatory and able to carry out work of a light or sedentary nature; HR, hazard ratio; OS, overall survival.

### Quality evaluation of the included studies

The quality evaluation of the seven included studies is shown in Supplementary Figure S1. Briefly, seven studies used random sequences; four studies used allocation concealment; six study subjects and operators used blinding; one study used outcome assessment blinding; all studies had complete data and no selective reporting. Overall, the included studies were of high quality.

### Meta-analysis results

**Figure 2:**
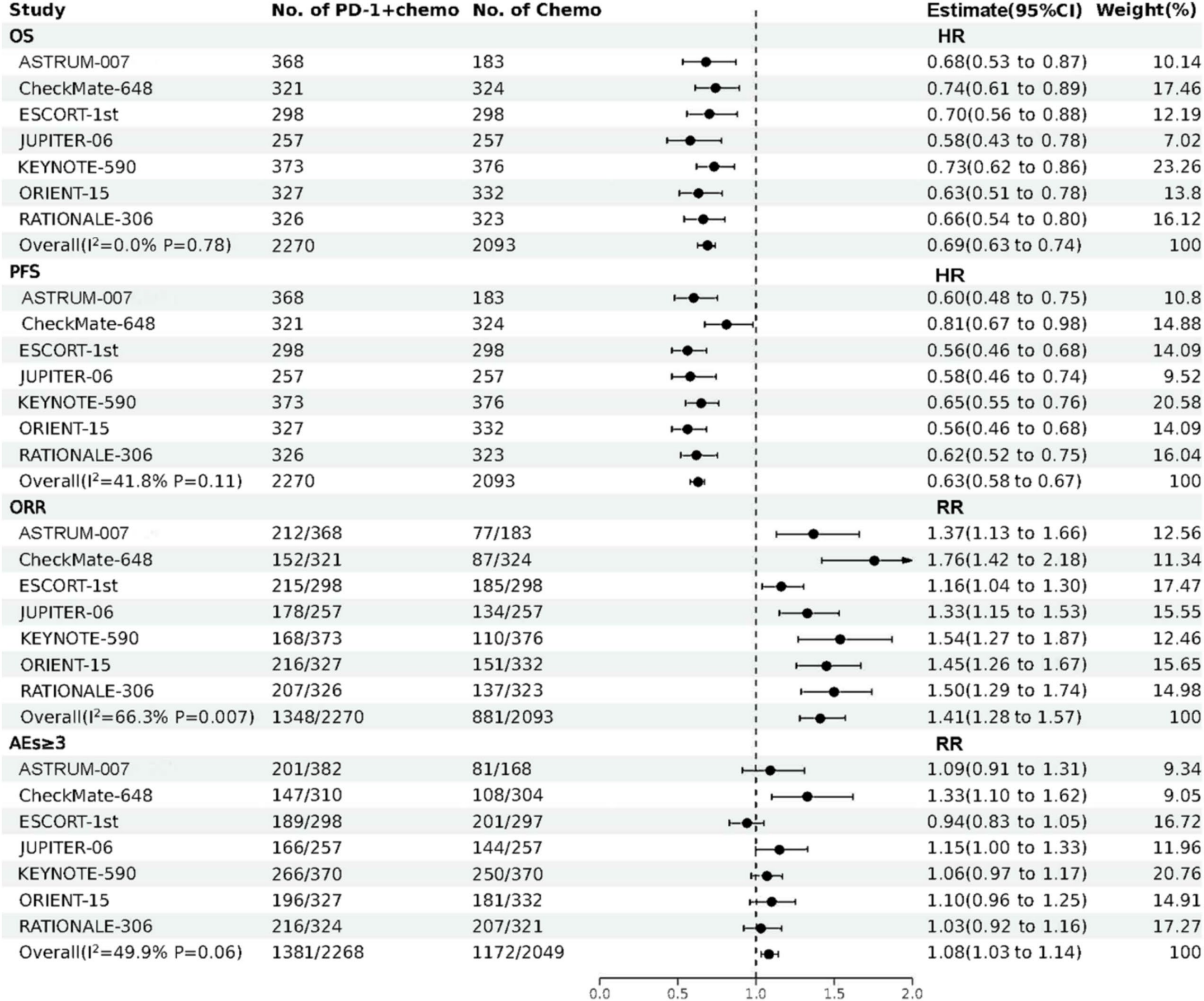
The forest plot about OS, PFS, ORR, and AEs (grade ≥ 3) of direct meta-analysis. PD-1 + chemo, PD-1 inhibitor combined with chemotherapy; Chemo, chemotherapy; CI, confidence interval; HR, hazard ratio; RR, risk ratio; OS, overall survival; PFS, progression free survival; ORR, objective response rate; AEs (grade ≥ 3), treatment related adverse events were greater than or equal to grade 3.

The forest plot about OS, PFS, ORR, and AEs (grade ≥ 3) of direct meta-analysis is shown in Figure 2. A network evidence plot for the included studies is shown in Supplementary Figure S2. And the forest plots about OS, PFS, ORR, and AEs (grade ≥ 3) of network meta-analyses are shown in Supplementary Figure S3.

**Figure 3:**
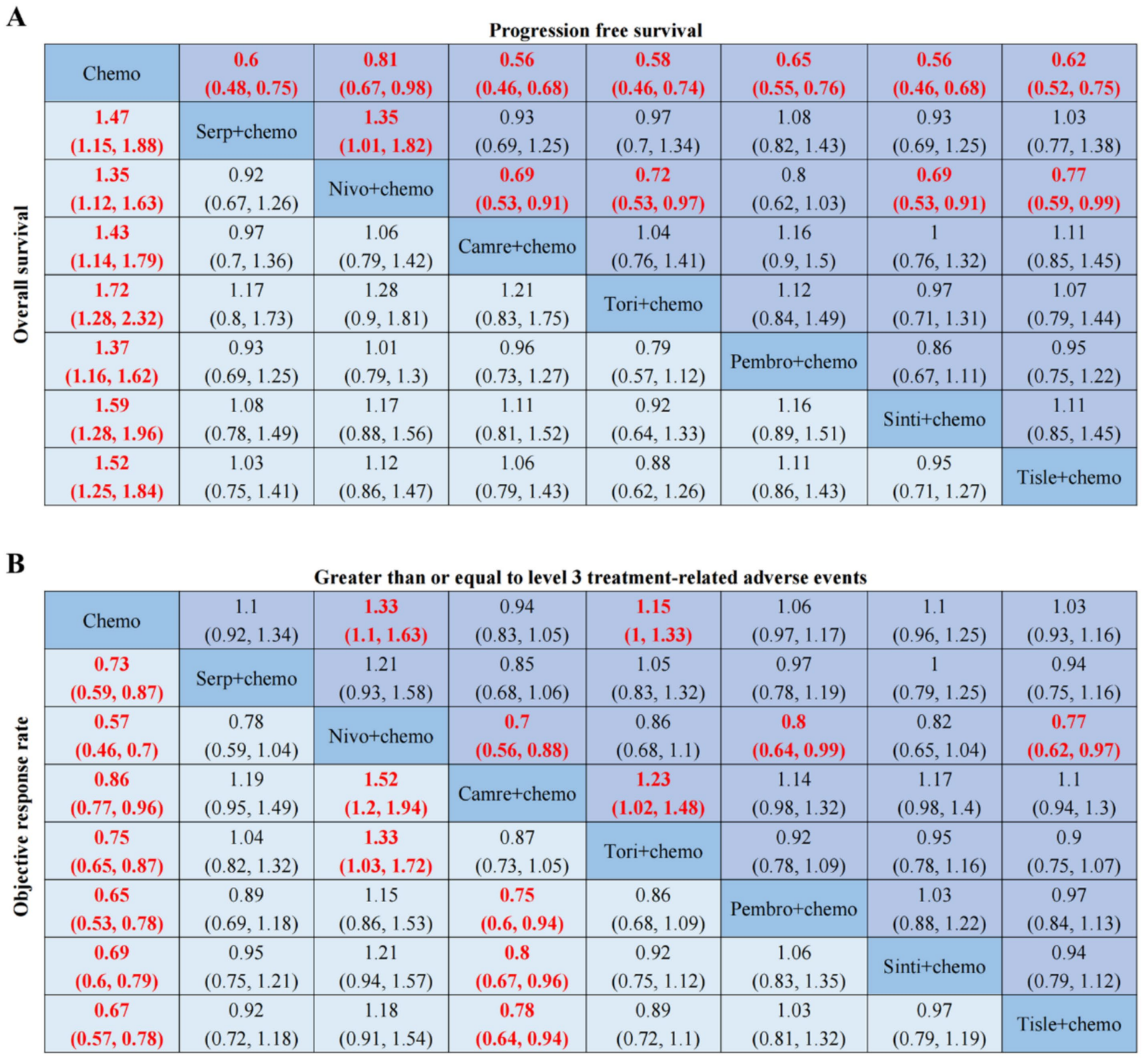
OS, PFS, ORR, AEs ≥ 3-related league tables. **(A)** HR 95%CIs for overall survival and progression free survival. **(B)** RR 95%CIs for objective response rate and greater than or equal to level 3 treatment-related adverse events. Chemo, chemotherapy; Serp, Serplulimab; Nivo, Nivolumab; Camre, Camrelizumab; Tori, Toripalimab; Pembro, Pembrolizumab; Sinti, Sintilimab; Tisle, Tislelizumab; HR, Hazard Ratio; RR, Risk Ratio; CI, confidence interval.

**Figure 4:**
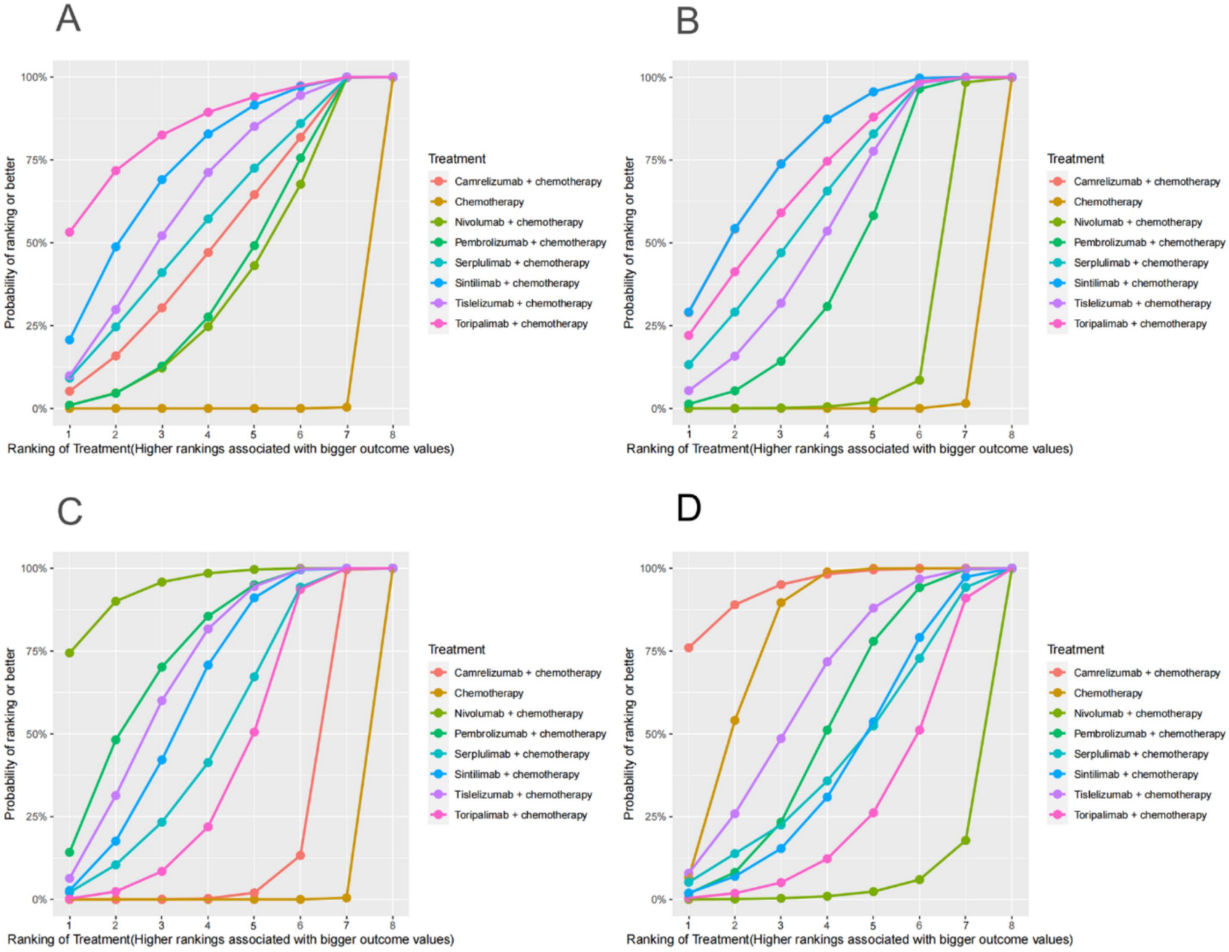
OS, PFS, ORR, AEs (grade ≥ 3)-related SUCRA. **(A)** SUCRA plot for overall survival. **(B)** SUCRA plot for progression free survival. **(C)** SUCRA plot for objective response rate. **(D)** SUCRA plot for treatment related adverse events were greater than or equal to grade 3. SUCRA, surface under the cumulative ranking curve.

**Figure 5:**
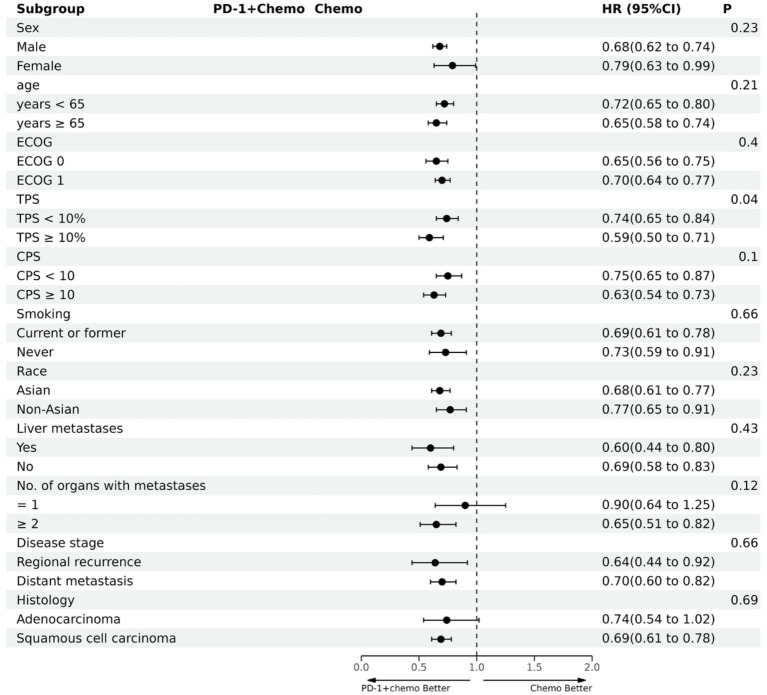
The forest plot of OS subgroup analysis. PD-1 + chemo, PD-1 inhibitor combined with chemotherapy; Chemo, chemotherapy; HR, hazard ratio; CI, confidence interval; P, represents the significance of differences between subgroups; ECOG, Eastern Cooperative Oncology Group; TPS, the tumor proportion score; CPS, the combined positive score.

PD-1 inhibitors combined with chemotherapy had a significant effect on OS, reducing the risk of death by 31% compared with chemotherapy (HR: 0.69, 95%CI: 0.63–0.74, *I*^2^ = 0.0%, *p* < 0.001). A network meta-analysis of the effects of seven PD-1 inhibitors combined with chemotherapy showed that: OS benefits, toripalimab combined chemotherapy (SUCRA = 0.84) > sintilimab combined chemotherapy (SUCRA = 0.73) > tislelizumab combined chemotherapy (SUCRA = 0.63) > serplulimab combined chemotherapy (SUCRA = 0.55) > camrelizumab combined with chemotherapy (SUCRA = 0.49) > pembrolizumab combined with chemotherapy (SUCRA = 0.39) > nivolumab combined with chemotherapy (SUCRA = 0.36) > chemotherapy (SUCRA = 0). The results of network meta-analysis in OS and the SUCRA are shown in Figures 3A, 4A, respectively. Subgroup analyses were performed based on gender, age, ECOG performance-status score, PD-L1 expression level TPS or CPS, and smoking status. Patients with TPS ≥ 10% had more significant OS improvement than those with TPS < 10% (Psubgroup = 0.04). The forest plot of OS subgroup analyses are shown in Figure 5.

**Figure 6:**
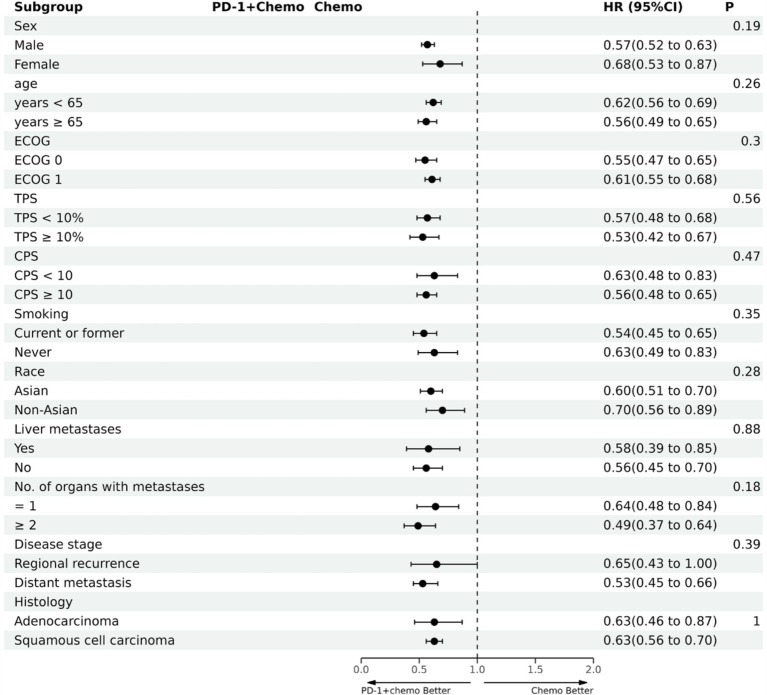
The forest plot of PFS subgroup analysis. PD-1 + chemo, PD-1 inhibitor combined with chemotherapy; Chemo, chemotherapy; HR, hazard ratio; CI, confidence interval; P, represents the significance of differences between subgroups; ECOG, Eastern Cooperative Oncology Group; TPS, the tumor proportion score; CPS, the combined positive score.

PD-1 inhibitors combined with chemotherapy were significantly effective in improving PFS, reducing the risk of death by 37% compared with chemotherapy (HR: 0.63, 95%CI: 0.58–0.67, *I*^2^ = 41.8%, *p* < 0.001). A network meta-analysis of the effects of seven PD-1 inhibitors combined with chemotherapy showed that: PFS benefits, sintilimab combined chemotherapy (SUCRA = 0.77) > camrelizumab combined chemotherapy (SUCRA = 0.77) > toripalimab combined chemotherapy (SUCRA = 0.69) > serplulimab combined chemotherapy (SUCRA = 0.62) > tislelizumab combined with chemotherapy (SUCRA = 0.55) > pembrolizumab combined with chemotherapy (SUCRA = 0.44) > nivolumab combined with chemotherapy (SUCRA = 0.16) > chemotherapy (SUCRA = 0). The results of network meta-analysis in PFS and the SUCRA are shown in Figures 3A, 4B, respectively. Subgroup analyses were conducted according to gender, age, ECOG performance-status score, TPS, CPS, and smoking status of patients, and the forest plot of PFS subgroup analysis is shown in Figure 6.

PD-1 inhibitors combined with chemotherapy had a significant effect on ORR. Compared with the chemotherapy group, PD-1 inhibitors were combined with chemotherapy (RR: 1.41, 95%CI: 1.28–1.57, *I*^2^ = 66.3%, *p* < 0.001). A network meta-analysis of the effects of seven PD-1 inhibitors combined with chemotherapy showed that: ORR improves results, nivolumab combined chemotherapy (SUCRA = 0.94) > pembrolizumab combined chemotherapy (SUCRA = 0.73) > tislelizumab combined chemotherapy (SUCRA = 0.68) > sintilimab combined chemotherapy (SUCRA = 0.61) > serplulimab combined chemotherapy (SUCRA = 0.48) > toripalimab combined chemotherapy (SUCRA = 0.40) > camrelizumab combined chemotherapy (SUCRA = 0.16) > chemotherapy (SUCRA = 0). The results of network meta-analysis in ORR and the SUCRA are shown in Figures 3B, 4C, respectively.

**Figure 7:**
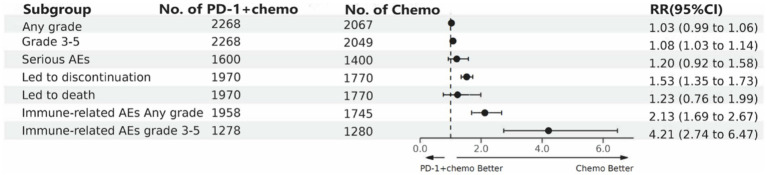
The forest plot of adverse events subgroup analysis. PD-1 + chemo, PD-1 inhibitor combined with chemotherapy; Chemo, chemotherapy; RR, risk ratio; CI, confidence interval; AEs; adverse events.

PD-1 inhibitors combined with chemotherapy increased the incidence of AEs (grade ≥ 3). Compared with the chemotherapy group, PD-1 inhibitors were combined with chemotherapy (RR: 1.08, 95%CI: 1.03–1.14, *I*^2^ = 49.9%, *p* = 0.002). A network meta-analysis of the incidence of AEs (grade ≥ 3) of seven PD-1 inhibitors combined with chemotherapy showed that: Camrelizumab combined chemotherapy (SUCRA = 0.94) > chemotherapy (SUCRA = 0.78) > tislelizumab combined chemotherapy (SUCRA = 0.63) > pembrolizumab combined chemotherapy (SUCRA = 0.51) > serplulimab combined chemotherapy (SUCRA = 0.42) > sintilimab combined chemotherapy (SUCRA = 0.41) > toripalimab combined chemotherapy (SUCRA = 0.27) > nivolumab combined chemotherapy (SUCRA = 0.04). The results of network meta-analysis in AEs (grade ≥ 3) and the SUCRA are shown in Figures 3B, 4D, respectively. The forest plot of AEs subgroup analysis is shown in Figure 7.

### Sensitivity analysis and publication bias

The sensitivity analyses of the included studies were carried out by the one-by-one exclusion method, and the results showed that there was no significant change in the results of our study after each study was excluded. Plots of sensitivity analyses for OS, PFS, ORR, and AEs (grade ≥ 3) are shown in Supplementary Figures S4A–D, respectively. Bias funnel plots were drawn for each outcome index, and Egger tests were also carried out. The results showed that *P*_(OS)_ = 0.06, *P*_(PFS)_ = 0.48, *P*_(ORR)_ = 0.02, *P*_(AEs ≥ 3)_ = 0.14. It suggests that there may exist publication bias in ORR indicator. Funnels of Publication bias for studies with OS, PFS, ORR, and AEs (grade ≥ 3) are shown in Supplementary Figures S5A–D, respectively.

## Discussion

This study conducted a comprehensive search in all of the available electronic databases, and seven RCTs were ultimately included. A direct meta-analysis was performed to assess the efficacy and safety of PD-1 inhibitors combined with chemotherapy for advanced EC. And then, a Bayesian network meta-analysis was conducted to explore the therapeutic difference in seven PD-1 inhibitors combined treatments. The results of a direct meta-analysis showed that PD-1 inhibitors combined chemotherapy significantly improved OS, PFS, and ORR in EC patients compared with chemotherapy alone, though they were associated with a higher incidence of immune-related AEs (grade ≥ 3). Due to the large sample size of all the included studies, the effect of small samples is almost no impact on the results of network meta-analysis. Especially, there is a lack of studies that directly compare the efficacy and safety of different PD-1 inhibitors combined with chemotherapy currently, so we conducted this network meta-analysis. The results of the network meta-analysis showed that toripalimab showed the best OS improvement, while sintilimab and camrelizumab exhibited superior PFS benefits. Nivolumab was associated with the highest ORR, and camrelizumab had the lowest incidence of severe AEs. We also conducted a sensitivity analysis on the main outcome indicators of direct meta-analysis; the results did not change significantly after each study was excluded, indicating that the results of our study were stable. According to the Egger tests, except for the study on ORR, no significant publication bias was found in other indicators. The publication bias in ORR may be caused by existing non-English publications that were not included in our study. So, these findings highlight the differential efficacy and safety profiles of PD-1 inhibitors in combination therapy, offering valuable insights for clinical decision-making.

In recent years, the development of PD-1 inhibitors has been very rapid, which means that previous meta-analyses need to be updated. Compared with the network meta-analysis of Gao et al. (25), our study has notable advantages: our study included more literature and comparisons; direct and indirect comparisons were performed, and systematic and comprehensive subgroup analyses were also conducted. At the same time, compared with the study of Li et al. (26), our study also has the following advantages: the number of included studies, sample-size, and drug types included is greater; sensitivity analyses and publication bias tests were performed on the results of the direct meta-analysis to make the conclusions more reliable. More importantly, the subgroup analysis in our study found different conclusions from Li et al. The study (26) concluded that patients with TPS ≥ 10% had a better OS improvement effect, and patients with CPS ≥ 10 had a better PFS advantage. However, our study only found that the difference in efficacy of PD-1 inhibitors combined with chemotherapy was related to the percentage of positive expression rate of PD-L1 in tumor cells. Patients with TPS ≥ 10% had more significant OS improvement than those with TPS < 10%. Our study included a larger sample size, and conducted more comprehensive comparisons, so the conclusions were more reliable. Clinically, PD-L1 expression is usually used to determine whether to treat tumors with PD-1 inhibitors. However, current studies on the use of TPS or CPS to evaluate the expression level of PD-L1 are highly controversial (27–30). Our study suggests that TPS is a better predictor of OS in EC patients treated with PD-1 inhibitors combined with chemotherapy. Our study can also provide a reference for research on the level of PD-L1 expression in EC patients.

PD-1 inhibitors show significant gender differences in the treatment of certain types of cancer. Previous study has shown that women with non-small cell lung cancer treated with PD-1 inhibitors have higher OS and remission rates than men, while in colorectal cancer patients, men have significantly prolonged OS (31). In addition, among patients with cutaneous melanoma, men treated with PD-1 inhibitors had a higher OS than women (32), but in our subgroup analysis on sex, gender differences were not factors affecting OS and PFS in patients treated with PD-1 inhibitors combined with chemotherapy for advanced EC. The efficacy of PD-1 inhibitors in the treatment of certain cancers is different in Asian and non-Asian patients. One meta-analysis suggested that PD-1 inhibitors were more effective in treating lung cancer in Asians than non-Asians (33), but our study did not find similar results. A meta-analysis of the effect of age factors on the efficacy of immunodetection point inhibitors in the treatment of advanced cancer showed that there was no significant correlation between the efficacy of PD-1 inhibitors and patient age (34), which is consistent with the conclusion of our study. In addition, relevant studies have shown that PD-1 inhibitors are more effective in treating squamous cell carcinoma than adenocarcinoma (35, 36), but our study did not find such a relationship. Therefore, the confounding factors, for example, gender, age, region or race, cancer type, etc., that may affect the efficacy of PD-1 inhibitors in the treatment of advanced EC need to be confirmed by more studies.

The therapeutic difference in OS or PFS among PD-1 inhibitors combined with chemotherapy may be related to the molecular structure of PD-1 inhibitors and the activation of additional immune cell pathways (37, 38). Toripalimab, a fully humanized IgG4, has a stronger binding affinity with PD-1 than other types of PD-1 inhibitors, such as nivolumab and pembrolizumab, and can further induce endocytotic action of the PD-1 receptor. The expression of PD-1 on the surface of the cell membrane was reduced (39, 40). This may be the reason for the better OS improvement in patients with toripalimab combined with chemotherapy compared to other PD-1 inhibitors combined with chemotherapy. Sintilimab and camrelizumab have a similar action effect as toripalimab. Thus, patients with sintilimab combined with chemotherapy and camrelizumab combined with chemotherapy have better PFS improvement compared to patients with other PD-1 inhibitors combined with chemotherapy (41). However, at present, there is a lack of direct comparative studies on the above combined schemes, so the above conclusions need to be verified by more high-quality and large-sample studies.

ORR differences among different PD-1 inhibitors combined with chemotherapy are related to the speed of the early tumor response induced by PD-1 inhibitors because ORR is an early benefit indicator for patients (42). Nivolumab can induce tumor responses faster than other PD-1 inhibitors, so nivolumab combined with chemotherapy has a high ORR. OS is an indicator of a patient’s long-term viability. Therefore, PD-1 inhibitors combined with chemotherapy may have a high ORR but a low OS. Therefore, clinical use needs to be based on the actual situation of patients.

We also performed a subgroup analysis of AEs, which showed no difference in the incidence of PD-1 inhibitor combined chemotherapy compared with chemotherapy alone for total AEs, severe AEs, or AEs resulting in death, but the incidence of PD-1 inhibitor combined chemotherapy was much higher than chemotherapy alone for immune-related AEs. The results of the network meta-analysis showed that camrelizumab, tislelizumab, pembrolizumab, serplulimab, and sintilimab combined chemotherapy did not differ significantly compared with chemotherapy alone in the incidence of AEs (grade ≥ 3), while nivolumab combined chemotherapy and toripalimab combined chemotherapy were significantly increased compared with chemotherapy alone in the incidence of AEs (grade ≥ 3), camrelizumab combined chemotherapy was low compared with other PD-1 inhibitor combined chemotherapy in the incidence of AEs (grade ≥ 3) and was lower than that of chemotherapy alone. It is worth noting that camrelizumab has obvious side effects of reactive capillary endothelial cell proliferation, and more attention should be paid to the monitoring and prevention of these side effects in clinical using (43). Therefore, in the clinical use of PD-1 inhibitors combined with chemotherapy, it is necessary to identify the appropriate cancer treatment population, conduct regular monitoring of patients, focus on drug combinations, and track and record adverse events to reduce the incidence of AEs in patients.

Our study also has some limitations. Network meta-analysis is susceptible to complex interactions and cannot completely replace direct comparative clinical trials. In different studies, the age, ECOG performance-status score, and proportion of PD-L1-positive patients were different. The types and doses of chemotherapy drugs used in the different studies were different. The included studies had smaller sample sizes in non-Asian EC patients and esophageal adenocarcinoma patients, and fewer studies were included when subgroup analysis was performed for certain factors. The included study (18), CheckMate-648, did not blind subjects or procedures. There are few studies on the relationship between OS, PFS, and PD-L1 expression levels. However, this study utilized the standard network meta-analysis approach and comprehensively analyzed the different therapeutic effects of PD-1 inhibitors combined with chemotherapy. The results of this study were robust. In addition, there has not been an investigation directly comparing the effects of PD-1 inhibitors combined with chemotherapy up until now. Therefore, our network meta-analysis provided reliable results from indirect comparisons, and the findings could provide proper references to clinical decision-makers.

In summary, PD-1 inhibitors combined with chemotherapy demonstrated superior efficacy over chemotherapy alone for advanced EC. Toripalimab, sintilimab or camrelizumab, nivolumab combined with chemotherapy might be the best in OS, PFS, and ORR, respectively. And camrelizumab combined with chemotherapy might have the lowest incidence of AEs (grade ≥ 3). Due to the limitations of the study, the conclusions need to be verified by RCTs with multi-center, high-quality, and large sample size.

## Data Availability

The original contributions presented in the study are included in the article/Supplementary material, further inquiries can be directed to the corresponding authors.
