# Supplementary material for: The efficacy and safety of PD-1 inhibitors combined with chemotherapy treatment for advanced esophageal cancer: a network meta-analysis
Source: Front Med (Lausanne). 2025 Jan 10;11:1515263. doi: 10.3389/fmed.2024.1515263 (PMC11759289; doi:10.3389/fmed.2024.1515263)
Supplement: Supplementary file 1 [file Data_Sheet_1.PDF]

Supplementary File S1. Detailed search strategies

PubMed:

#1 "Immune Checkpoint Inhibitors"[Mesh]

#2 "Immune Checkpoint Inhibitor" OR "immune check blockade" OR PD-1 OR PD-L1 OR CTLA-4 OR "nivolumab"[Mesh] OR nivolumab OR MDX-1106 OR MDX1106 OR "MDX 1106" OR Opdivo OR BMS-936558 OR BMS936558 OR "BMS 936558" OR ONO-4538 OR ONO4538 OR "ONO 4538" OR "pembrolizumab"[Supplementary Concept] OR pembrolizumab OR MK-3475 OR Keytruda OR lambrolizumab OR SCH-900475 OR "camrelizumab"[Supplementary Concept] OR camrelizumab OR SHR-1210 OR "SHR 1210" OR carrelizumab OR "sintilimab"[Supplementary Concept] OR sintilimab OR "IBI 308" OR IBI308 OR IBI-308 OR "toripalimab"[Supplementary Concept] OR toripalimab OR "tislelizumab"[Supplementary Concept] OR tislelizumab OR BGB-A317 OR "atezolizumab"[Supplementary Concept] OR atezolizumab OR anti-PDL1 OR MPDL3280A OR MPDL-3280A OR Tecentriq OR RG7446 OR RG-7446 OR "durvalumab"[Supplementary Concept] OR durvalumab OR MEDI4736 OR MEDI-4736 OR Imfinzi OR "avelumab"[Supplementary Concept] OR avelumab OR MSB0010718C OR MSB-0010718C OR bavencio OR MSB-0010682 OR MSB0010682 OR "ipilimumab"[Mesh] OR ipilimumab OR "Anti-CTLA-4 MAb Ipilimumab" OR Anti "CTLA 4 MAb Ipilimumab" OR "Ipilimumab, Anti-CTLA-4 MAb" OR "MDX 010" OR MDX-010 OR MDX010 OR MDX-CTLA-4 OR "MDX CTLA 4" OR Yervoy OR "tremelimumab"[Supplementary Concept] OR tremelimumab OR CP-675,206 OR "CP 675206" OR CP-675206 OR CP675206 OR "CP 675" OR CP-675 OR "CP675 cpd" OR Ticilimumab OR serplulimab

#3 #1 OR #2

#4 "Drug Therapy"[Mesh]

#5 "Therapy, Drug" OR "Drug Therapies" OR "Therapies, Drug" OR Chemotherapy OR Chemotherapies OR Pharmacotherapy OR Pharmacotherapies

#6 #4 OR #5

#7 "Esophageal Neoplasms"[Mesh]

#8 "Esophageal Neoplasm" OR "Esophagus Neoplasm" OR "Esophagus Cancer" OR "Esophageal Cancer" OR "Esophageal Squamous Cell Cancer" OR "Esophageal

Carcinoma"

#9 #7 OR #8

#10 #3 AND #6 AND #9

Embase:

#1 'immune checkpoint inhibitor'/exp OR 'immune check blockade' OR 'pd 1' OR 'pd l1' OR 'ctla 4' OR nivolumab OR MDX-1106 OR MDX1106 OR nivolumab OR MDX-1106 OR MDX1106 OR 'MDX 1106' OR Opdivo OR BMS-936558 OR BMS936558 OR 'BMS 936558' OR ONO-4538 OR ONO4538 OR 'ONO 4538' OR OR pembrolizumab OR MK-3475 OR Keytruda OR lambrolizumab OR SCH-900475 OR camrelizumab OR SHR-1210 OR 'SHR 1210' OR carrelizumab OR sintilimab OR 'IBI 308' OR IBI308 OR IBI-308 OR toripalimab OR tislelizumab OR BGB-A317 OR atezolizumab OR anti-PDL1 OR MPDL3280A OR MPDL-3280A OR Tecentriq OR RG7446 OR RG-7446 OR durvalumab OR MEDI4736 OR MEDI-4736 OR Imfinzi OR avelumab OR MSB0010718C OR MSB-0010718C OR bavencio OR MSB-0010682 OR MSB0010682 OR ipilimumab OR 'Anti-CTLA-4 MAb Ipilimumab' OR Anti 'CTLA 4 MAb Ipilimumab' OR 'Ipilimumab, Anti-CTLA-4 MAb' OR 'MDX 010' OR MDX-010 OR MDX010 OR MDX-CTLA-4 OR 'MDX CTLA 4' OR Yervoy OR tremelimumab OR CP-675,206 OR 'CP 675206' OR CP-675206 OR CP675206 OR 'CP 675' OR CP-675 OR 'CP675 cpd' OR Ticilimumab OR serplulimab

#2 'drug therapy'/exp OR 'therapy, drug' OR 'drug therapies' OR 'therapies, drug' OR chemotherapy OR chemotherapies OR pharmacotherapy OR pharmacotherapies

#3 'esophageal neoplasms'/exp OR 'esophageal neoplasm' OR 'esophagus neoplasm' OR 'esophagus cancer' OR 'esophageal cancer' OR 'esophageal squamous cell cancer' OR 'esophageal carcinoma'

#4 #1 AND #2 AND #3

Cochrane Library:

#1 ("Immune Checkpoint Inhibitors") OR ("Immune Checkpoint Inhibitor") OR (PD-1) OR (PD-L1) OR (CTLA-4) OR ("immune check blockade")

#2 (nivolumab) OR (pembrolizumab) OR (camrelizumab) OR (sintilimab) OR (toripalimab)

#3 (tislelizumab) OR (atezolizumab) OR (durvalumab) OR (avelumab) OR (ipilimumab)

#4 (tremelimumab) OR (serplulimab)

#5 ("Drug Therapy") OR ("Therapy, Drug") OR ("Drug Therapies") OR ("Therapies, Drug") OR (Chemotherapy)

#6 (Chemotherapies) OR (Pharmacotherapy) OR (Pharmacotherapies)

#7 ("Esophageal Neoplasms") OR ("Esophageal Neoplasm") OR ("Esophagus Neoplasm") OR ("Esophagus Cancer") OR ("Esophageal Cancer")

#8 ("Esophageal Squamous Cell Cancer") OR ("Esophageal Carcinoma")

#9 #1 OR #2 OR #3 OR #4

#10 #5OR #6

#11 #7 OR #8

#12 #9 AND #10 AND #11

Web of Science

#1 "Immune Checkpoint Inhibitors" (Topic) or "immune check blockade" (All Fields) or PD-1 (All Fields) or PD-L1 (All Fields) or CTLA-4 (All Fields) or nivolumab (All Fields) or pembrolizumab (All Fields) or camrelizumab (All Fields) or toripalimab (All Fields) or atezolizumab (All Fields) or durvalumab (All Fields) or avelumab (All Fields) or ipilimumab (All Fields) or tremelimumab (All Fields) or serplulimab (All Fields)

#2 "Drug Therapy" (Topic) or "Drug Therapies" (All Fields) or "Drug Therapies" (All Fields) or Chemotherapies (All Fields) or Pharmacotherapy (All Fields) or Pharmacotherapies (All Fields) or Chemotherapy (All Fields)

#3 "Esophageal Neoplasms" (Topic) or "Esophagus Neoplasm" (All Fields) or "Esophagus Cancer" (All Fields) or "Esophageal Cancer" (All Fields) or "Esophageal Squamous Cell Cancer" (All Fields) or "Esophageal Carcinoma" (All Fields)

#4 #1 AND #2 AND #3

Scopus

#1 (TITLE-ABS-KEY ("Immune Checkpoint Inhibitors") OR TITLE-ABS-KEY ("Immune Checkpoint Inhibitor") OR TITLE-ABS-KEY (pd-1) OR TITLE-ABS-KEY (pd-l1) OR TITLE-ABS-KEY (ctla-4) OR TITLE-ABS-KEY (nivolumab OR mdx-1106 OR mdx1106 OR "MDX

1106" OR opdivo OR bms-936558 OR bms936558 OR "BMS 936558" OR ono-4538 OR ono4538 OR "ONO 4538" OR pembrolizumab OR mk-3475 OR keytruda OR lambrolizumab OR sch-900475 OR camrelizumab OR shr-1210 OR "SHR 1210" OR carrelizumab OR sintilimab OR sintilimab OR "IBI 308" OR ibi308 OR ibi-308 OR "toripalimab OR toripalimab OR " tislelizumab OR tislelizumab OR bgb-a317 OR atezolizumab OR atezolizumab OR anti-pdl1 OR mpdl3280a OR mpdl-3280a OR tecentriq OR rg7446 OR rg-7446 OR durvalumab OR durvalumab OR medi4736 OR medi-4736 OR imfinzi OR avelumab OR avelumab OR msb0010718c OR msb-0010718c OR bavencio OR msb-0010682 OR msb0010682 OR ipilimumab OR ipilimumab OR "Anti-CTLA-4 MAb Ipilimumab" OR anti "CTLA 4 MAb Ipilimumab" OR "Ipilimumab, Anti-CTLA-4 MAb" OR "MDX 010" OR mdx-010 OR mdx010 OR mdx-ctla-4 OR "MDX CTLA 4" OR yervoy OR "tremelimumab OR tremelimumab OR CP-675,206 OR " cp 675206 " OR CP-675206 OR CP675206 OR " cp 675 " OR CP-675 OR " cp675 AND cpd " OR ticilimumab OR serplulimab))

#2 (TITLE-ABS-KEY ("drug therapy") OR TITLE-ABS-KEY ("therapy, drug" OR "drug therapies" OR "therapies,drug" OR chemotherapy OR chemotherapies OR pharmacotherapy OR pharmacotherapies))

#3 (TITLE-ABS-KEY ("esophageal neoplasms") OR TITLE-ABS-KEY ("esophageal neoplasm" OR "esophagus neoplasm" OR "esophagus cancer" OR "esophageal cancer" OR "esophageal squamous cell cancer" OR "esophageal carcinoma"))

#4 #1 AND #2 AND #3
