# Supplementary figures and images for: The efficacy and safety of PD-1 inhibitors combined with chemotherapy treatment for advanced esophageal cancer: a network meta-analysis
Source: Front Med (Lausanne). 2025 Jan 10;11:1515263. doi: 10.3389/fmed.2024.1515263 (PMC11759289; doi:10.3389/fmed.2024.1515263)

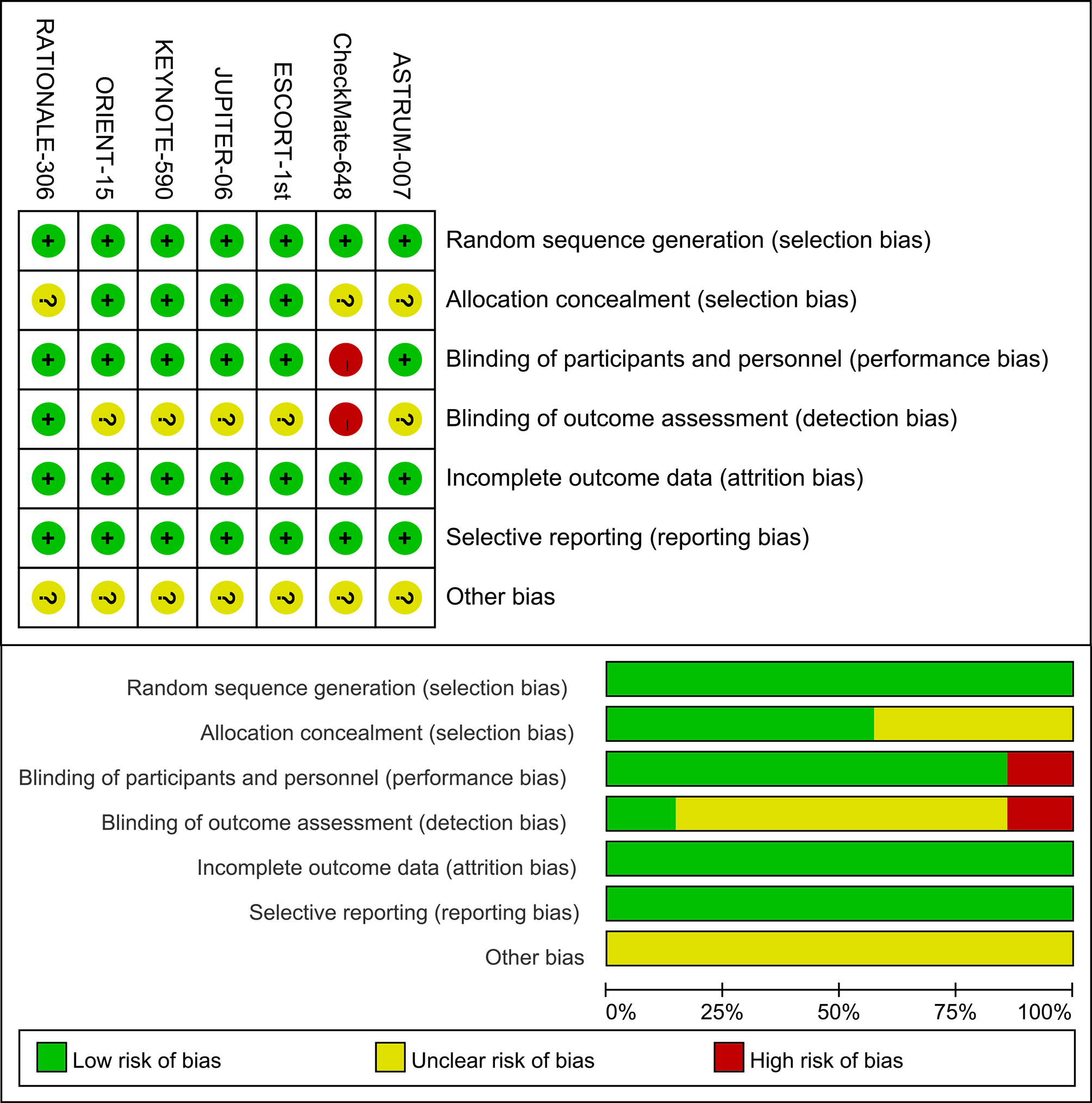

Supplement: Supplementary file 2 [file Image_1.TIF]

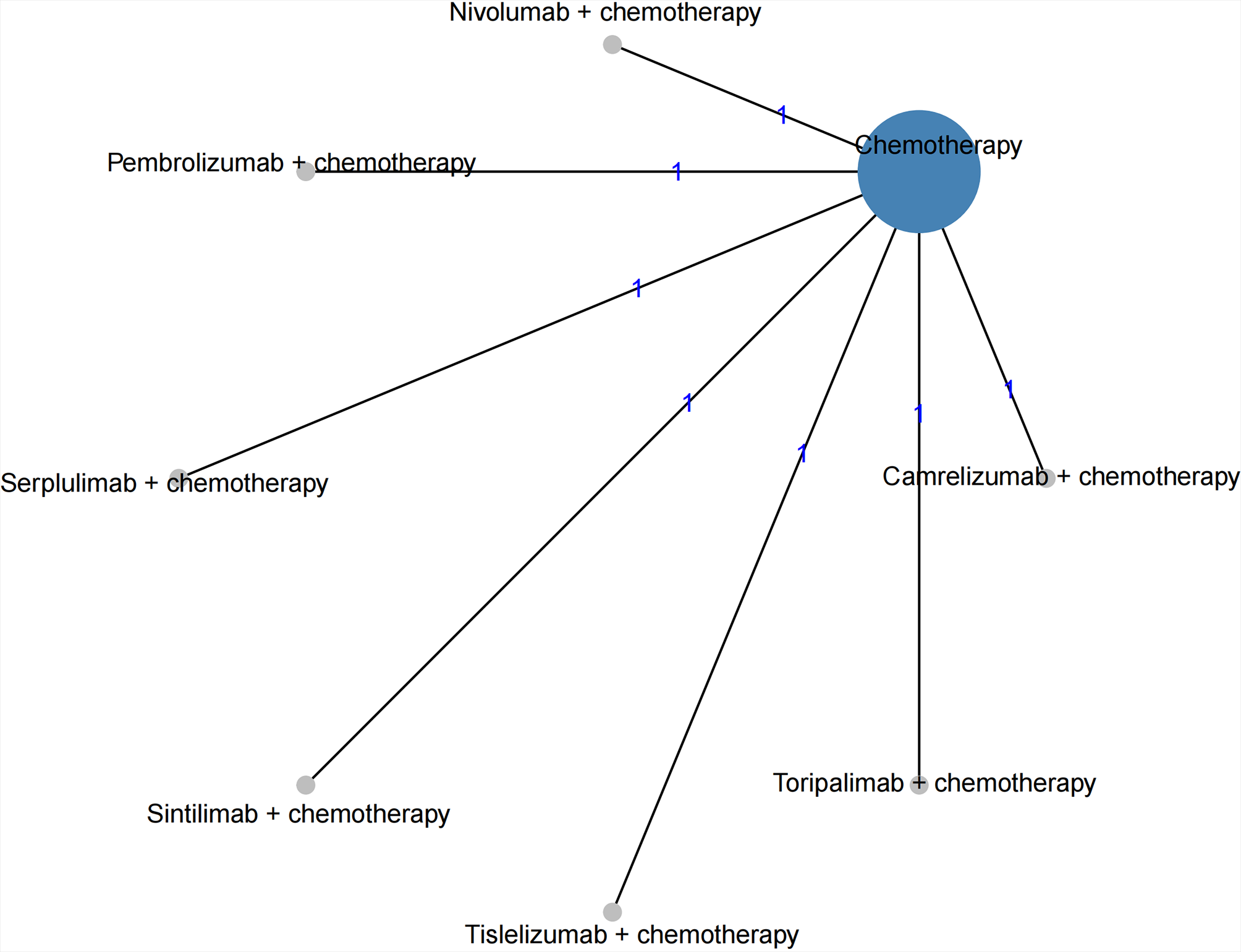

Supplement: Supplementary file 3 [file Image_2.TIF]

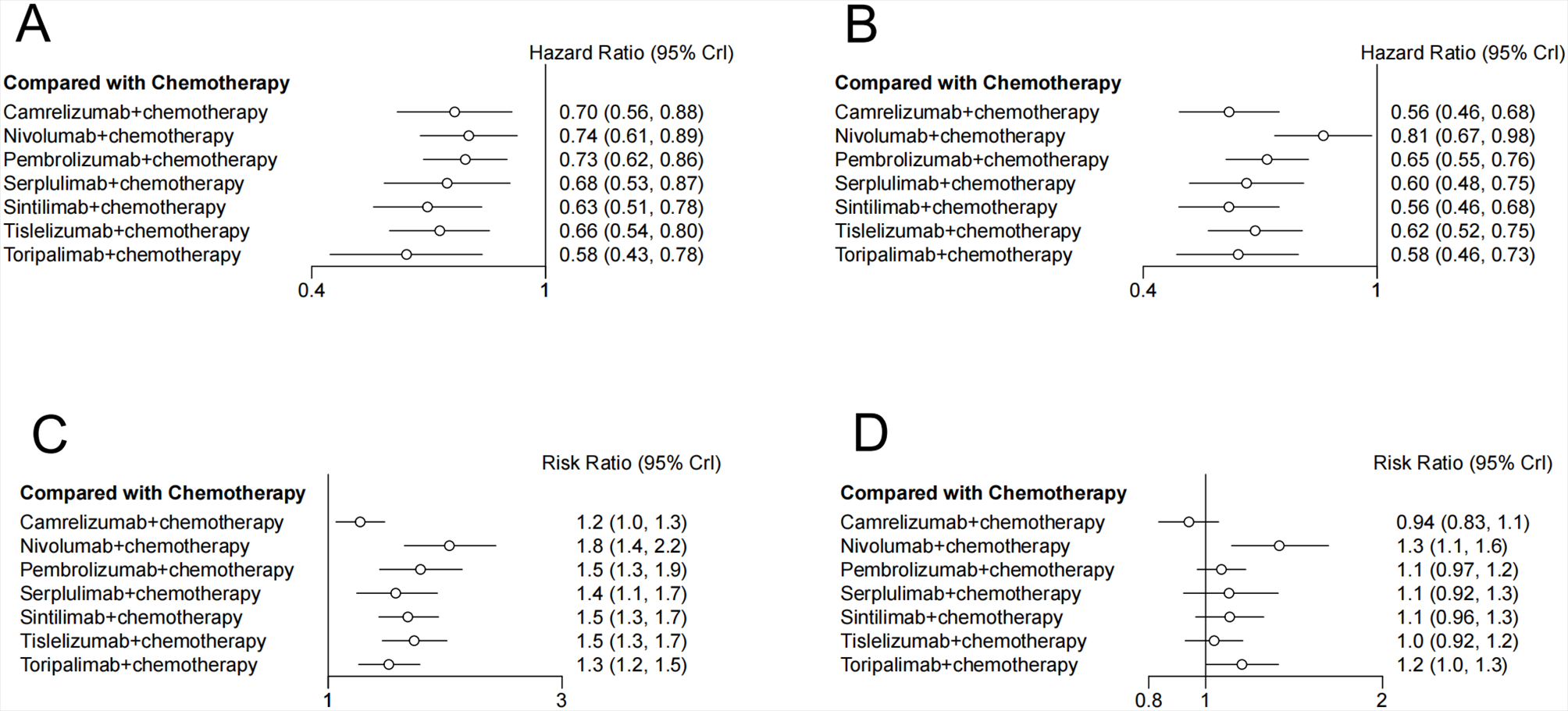

Supplement: Supplementary file 4 [file Image_3.TIF]

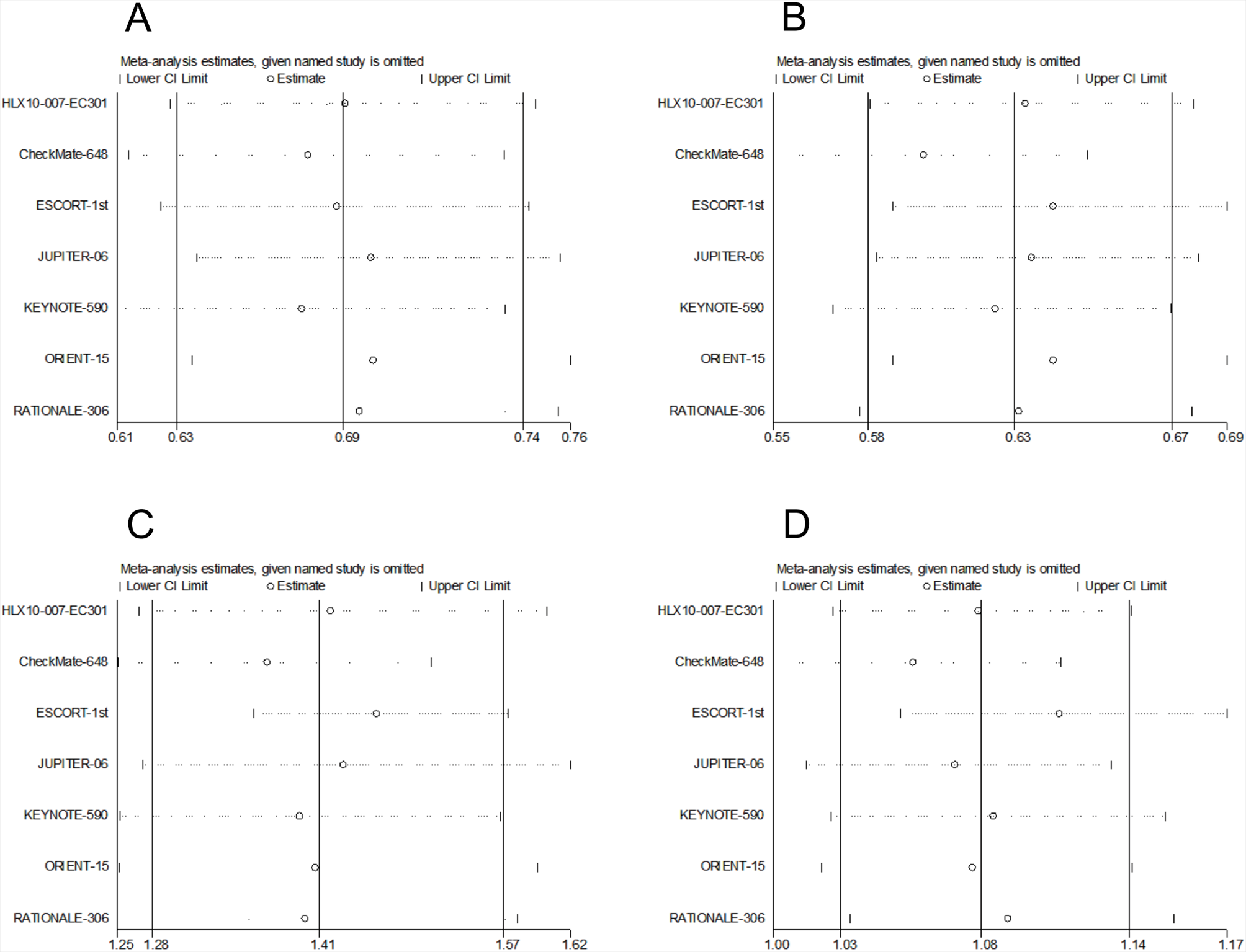

Supplement: Supplementary file 5 [file Image_4.TIF]

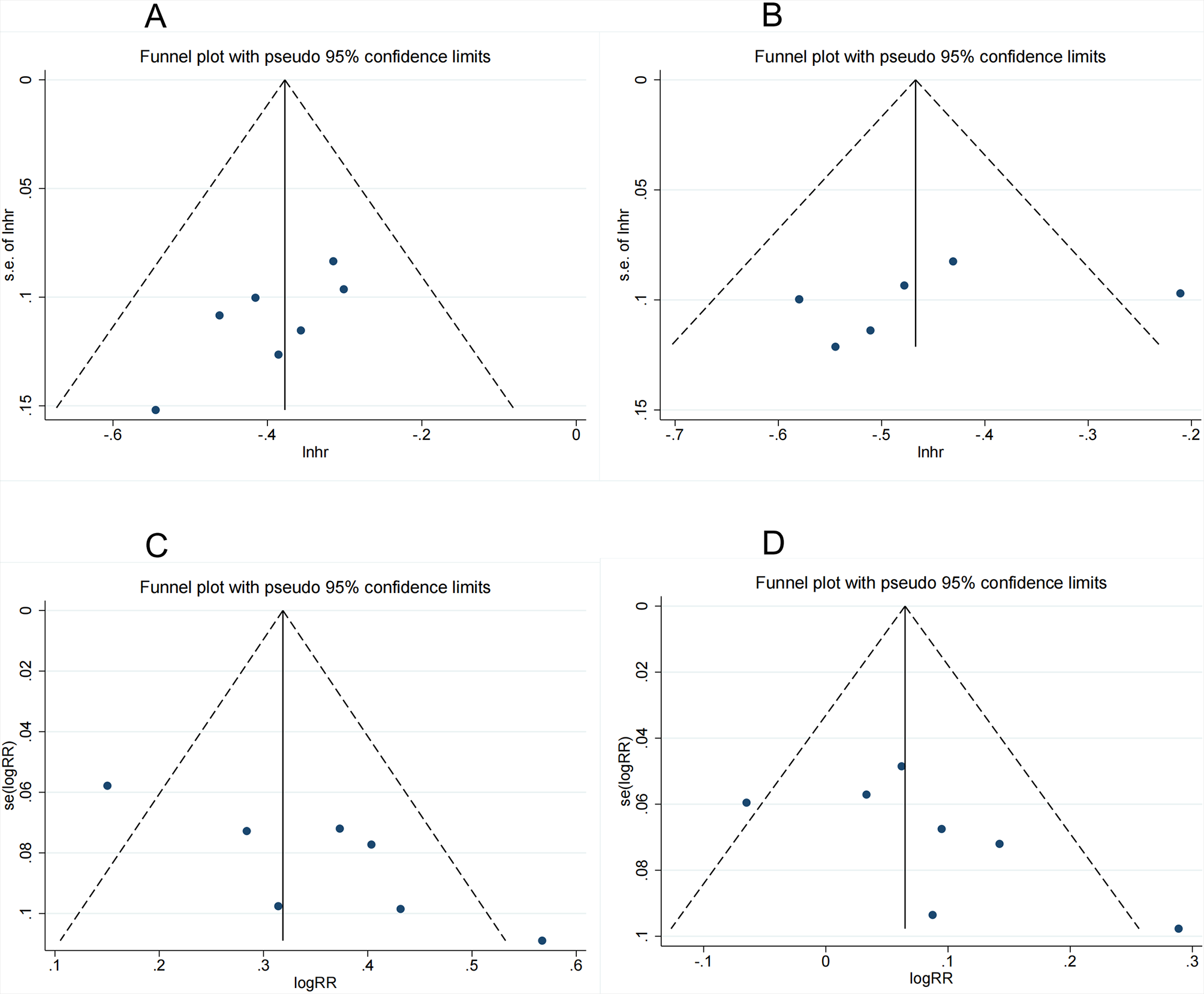

Supplement: Supplementary file 6 [file Image_5.TIF]
